# Genotoxicity and mutagenicity research in Quilombola communities

**DOI:** 10.1038/s41598-020-71195-4

**Published:** 2020-08-26

**Authors:** Aroldo Vieira de Moraes Filho, João Antonio Xavier Manso, Wanderléia Eleutério Martins, Núbia Aguiar Marinho, Mônica de Oliveira Santos, José Perim Neto, Sabrina Sara Moreira Duarte, Aparecido Divino da Cruz, Cláudio Carlos da Silva, Mônica Santiago Barbosa, Débora de Jesus Pires, Lílian Carla Carneiro

**Affiliations:** 1 Graduate Program in Health Sciences, Federal University of Goiás, Institute of Tropical Pathology and Public Health, Biotechnology Laboratory of Microorganisms, Federal University of Goiás, Goiânia, Goiás Brazil; 2 Replicon Research Center, Master’s Program in Genetics, School of Agrarian and Biological Sciences, Pontifical Catholic University of Goiás, Goiânia, Goiás Brazil; 3 Goiás State University, Itumbiara University Unit, Itumbiara, Goiás Brazil

**Keywords:** Genetics, Biomarkers

## Abstract

The Quilombola communities are mostly isolated and deprived of sources of treated water, garbage collection and sewage, consuming fresh water from wells, streams, lakes, among others. This lack of basic infrastructure can be a relevant factor in exposing residents to substances and factors that are harmful to the integrity of their genetic material that can lead to carcinogenesis. Based on this, the objective of this study was to evaluate the genomic and mutagenic/cytotoxic damage in the adult population of two Quilombola communities (one urban and another rural region), in the state of Goiás, Brazil. For this purpose, the leukocyte of peripheral blood Comet Assay in 68 individuals and Micronucleus Test from exfoliated buccal cells of oral mucosa in 21 volunteers were performed. The results evidenced genomic damage, especially for the community of Aparecida de Goiânia city, which detected significant values (*p* < 0.05), for the length of the comet’s tail and for of the Olive Tail Moment. In the micronucleus test, significant differences were only detected (*p* < 0.05), when it came to the distribution of nuclear changes among the groups. Therefore, it is essential to perform constant population biomonitoring studies to help guarantee health and, consequently, the quality of life.

## Introduction

In Brazil, “Quilombo” means any group of black people, living together, with subsistence autonomy, that did not were freed of slave condition^1^. In 1,850, Brazil's written and plowed land law reclassified the term Quilombola by classifying the people as Africans descendants into the category of “freed”, but excluding them from being Brazilian^2^.

Quilombola communities are mostly isolated and deprived of sources of treated water, garbage collection and sewage, consuming fresh water from wells, streams, lakes, among others. This lack of basic infrastructure can be a relevant factor in the exposure of its residents to substances and factors harmful to the integrity of their genetic material^3^.

In the health area, interest in quality of life has influenced public policies and practices, with the understanding that health involves all aspects of an individual's life. Therefore, the conditions of the health/disease process are multifactorial, if they are related to social, economic, psychological and environmental, life experiences and worldview of each individual^4–6^.

People are often exposed to environmental agents that can induce changes at both cellular and molecular levels, including mutations. These lesions may be caused by mutagenic agents (chemical, physical or biological) and may be harmful to cells^7^.

Thus, analyzes of genotoxicity, cytotoxicity and mutagenicity through the Comet assay and the Micronucleus (MN) test, can contribute to the research of quality of life and the prevention of possible diseases resulting from this environmental toxicity, in which these communities are in daily contact^8^. These two tests are the most used for the evaluation of genotoxicity in population studies. The comet assesses DNA damage before going through the repair mechanism, so it is repairable damage. On the other hand, the MN test assesses mutations (fixed lesions) in DNA^9^.

Thus, considering the impact of limited infrastructure on the quality of life of people from poor and/or isolated communities, this study aimed to infer and describe the profile of genotoxic, cytotoxic and mutagenic damage in a sample of individuals from two quilombola communities located in the Goiás, Brazil, using the Comet and Micronucleus tests.

## Material and methods

### Ethical terms

The research was conducted after approval by the Research Ethics Committee of the Federal University of Goiás CEP/UFG (Protocol number: 65338017.6.0000.5083) approved in 04/07/2017. All procedures were performed according to the guidelines of the National Health Council Resolutions, under 466/2012 and 510/2016 legislation^10^.

Samples blood (5 mL) and samples of exfoliated buccal cells (in duplicate), collected with a spatule, were collected in the two communities studied, including the Quilombolas aged older than eighteen and who signed the Informed Consent Form (ICF).

### Study population

The participating Quilombola communities, are located at Jardim Cascata in the municipality of Aparecida de Goiânia city, Goiás State, (urban community), on coordinates (16°49ʹ35ʺ S and 49°19ʹ06ʺ W) and in the rural area of Silvânia city, Goiás State, (rural community), near coordinates (16°41ʹ18ʺ S and 48°15′23″ W).

The control group consisted of individuals segregated from Quilombola communities, from the Goiânia city Metropolitan Region. A screening was carried out by interviewing volunteers with lower marital age. According with lifestyle, smokers and people who used alcohol were exclusion criteria. After the first contact and explanation of the project, the individuals who agreed to participate, signed the informed consent form and were instructed regarding the preparation and fasting needed in the hours before the collection of blood and mucosa samples.

A number de 100 nucleoids per individual were counted a and, the results averaged for each parameter. Fifteen participants from the control group, 25 participants from the Quilombola community of Aparecida de Goiânia city (urban community), and 29 participants from the Quilombola community of Silvânia city (rural community), were evaluated, according to the availability of the groups studied.

### Comet assay

The protocol used was proposed by Singh et al.^11^, and Silva and Cruz^12^, with modifications. During the methodology, the samples were handled at 4 °C temperature, protected from light. Approximately 10 µL of the whole blood sample was soaked in 120 µL at low melting point agarose − 0.75% concentration. The mixture was placed on Normal Melting Point agarose coated glass slide, at − 1.5% concentration. After solidification in the refrigerator, cells were lysed with a lysis solution (pH 10), containing Sodium Chloride (NaCl), 100 mM Ethylenediamine Tetraacetic Acid (EDTA), 10 mM Tris, 1% Tween 80 and 10% dimethyl sulfoxide (DMSO) overnight.

After lyses, the slides were placed in horizontal electrophoresis unit for nucleic acid separations. To allow DNA separation, the slides were incubated for 25 min in alkaline solution for electrophoresis, made at the time of use, containing 300 mM Sodium Hydroxide (NaOH) and 1 mM EDTA, pH > 13 at 4 °C temperature. The slides were subjected to an electric current of 300 mA at 1 V/cm for 25 min. After electrophoresis experiment, the slides were neutralized with 0.4 M Tris Base (pH = 7.5), then washed with distilled water, fixed in absolute ethanol and stored at room temperature. Slide staining occurred moments before analysis by depositing 20 μL (20 μg/mL) of the ethidium bromide staining solution, followed by the addition of cover slips.

Slides were analyzed under epifluorescence microscopy using 515–560 nm excitation filter set and 590 nm barrier filter for red fluorescence. The cores were visualized at 400× and 600× magnification and the fluorescent images were captured and exported in bitmap extension using the ZEN lite software, version 2.6, Blue Edition (Carl Zeiss, Germany). Subsequently, they were analyzed using the Comet Score 2.0 software, following the parameters: Tail Length, percentage of DNA at the Olive Tail Moment (OTM), was considered the most relevant parameter^13,14^.

The data were subjected to the normality test (Kolmogorov–Smirnov), but did not present a normal distribution. Thus, the Kruskal–Wallis test was used for non-parametric analysis of variance at 5% significance level (p < 0.05). Dunn's method was used, when necessary. Statistical analyzes were performed using the BioEstat 5.0 software.

### Micronucleus test

The protocol used was proposed by Souto et al.^15^, with modifications. Prior to the collection of exfoliated buccal cells, the participants were asked to rinse their mouth with running water to eliminate the presence of possible food scraps and to decrease the bacterial concentration in the smear slides. Using the slides properly cleaned with 99.5° GL alcohol, and a water-moistened tongue depressor (spatule), the exfoliated buccal cells were scraped and spread over the slides. The smear was made at room temperature (~ 25 °C) and then fixed in alcohol 99.5° GL for 15 min.

Hydrolysis was performed at a titer of 1:10 (10%), using 20 mL hydrochloric acid (HCl) and 200 mL distilled water. The indicated volume was sufficient for 10 slides. In order to prepare the solution, the acid was slowly added to decrease evaporation. The slides were left in 10% HCl solution, for 2 min, at room temperature and then for 6 min, at 60 °C temperature. Lastly, the slides were taken back to room temperature.

The slides were dipped in basic fuchsin solution for 15 min in the dark and then rinsed lightly with water to remove excess dye. The slides were then brought to the Fast Green solution for 10 s. After time, the slides were rinsed in 70% alcohol.

Slides with dye deposits, glove residues and other materials, after staining were run through a battery of 95% ethanol for one minute, butanol for one minute, butanol/xylene (50:50 mixtures) for 1 min and xylene, until assembled with Canada balm. After this stage the cells were analyzed under the 100 × objective optical microscope.

For micronucleus analysis, 500 cells per slide and two slides per individual were quantified, thus 1,000 cells per individual. The micronuclei and other changes were determined using the following criteria: size smaller than 1/3 of the nucleus; same focus plane of the nucleus; pattern of chromatin structure and color identical to that of the nucleus; no connection with the nucleus (micronucleus). Seven volunteers were evaluated in each of the groups studied (Quilombola communities plus the control group), totaling 21 individuals.

The changes counted were: micronucleus, “Broken Egg”, trinucleated, binucleated cells, cariorhexis, apoptosis, budding, karyolysis and nucleoplasmic bridge.

Statistical analysis was performed using Bioestat 5.0 software, whereby the data were submitted to normality tests (Shapiro–Wilk or Kolmogorov–Smirnov), as demanded. Subsequently, micronucleus rates and nuclear alterations between groups were inferred separately through analysis of variance. The ANOVA (analysis of variance) and Kruskal–Wallis tests were used, as required. All tests were applied at 5% significance level (p < 0.05).

### Ethics approval

The research was conducted after approval by the Research Ethics Committee of the Federal University of Goiás CEP/UFG (Protocol number: 65338017.6.0000.5083), approved on 04/07/2017.

### Consent to participate

After the first contact and explanation of the project, the individuals who agreed to participate, signed the informed consent form and were instructed regarding the preparation and fasting needed in the hours before the collection of blood and mucosa samples.

## Results

### Comet assay

The normality analysis showed nonparametric distributions for the parameters analyzed in comet assay. Thus, Kruskal–Wallis test in conjunction with the Dunn method was used to infer the data (Table 1).

**Table 1:** Mean and standard deviation and *p* value of the parameters analyzed in the comet assay.

Groups (N)	Mean ± SD		
	Tail length	% of DNA in the tail	OTM
Control (15)	1.6 ± 0.7^a^	6.7 ± 2.8	0.3 ± 0.2^a^
Urban Zone (24)	14.2 ± 12.3^b^	10.3 ± 7.7	3.1 ± 4.9^b^
Rural Zone (29)	8.0 ± 5.4^b^	8.7 ± 6.5	1.2 ± 1.8^ab^
*p* value	< 0.01	0.47	< 0.01

*SD* standard deviation, *OTM* Olive Tail Moment, *p* probability of differences (or effect) observed among groups being due chance; p value = Kruskal–Wallis p value; ^a,b and ab^ = symbols arbitrarily assigned in the differentiation by Dunn's method; Urban Zone = community quilombola of Aparecida de Goiânia city; Rural Zone = community quilombola of Silvania city. Source: Own data.

Significant differences were detected for Tail Length parameter (p < 0.05). Substantially lower values were obtained to the control group, there is a significant difference among control group and test group. The damage contrast among the groups, suggests that the quilombola communities are under the effect of some genotoxic factor, due to the longer tail of the comets in these groups samples.

The data from the comet assay for the OTM also showed significant differences (p < 0.05). Differences in mean between the control group and the urban quilombola community (Aparecida de Goiânia city) were observed, indicating genotoxic activity. The rural quilombola of Silvania community presented an average close to the control group and studied community and no significant differences were detected (p > 0.05). It is suggested that this result is due to the percentage of DNA in the tail, which was not significant among the groups evaluated (p > 0.05), but which constitutes a requirement for calculating the OTM.

The DNA repair has been detected in longitudinal approaches^16,17^_._ It is conjectured that the DNA of individuals from the Silvania quilombola community could be under the effect of a frequent repair mechanism, due to exposure chronic (continuous exposure), which would possibly hide the real genomic damage. It should be noted that this is a rural population, usually in contact with pesticides, assuming that this could also apply to the quilombola community of Aparecida de Goiânia city, considering a less continuous DNA repair mechanism.

### Micronucleus test

A mutagenicity assessment was carried out among the two quilombola communities and a control group (total of 3 groups) using the micronucleus test (MN). Seven people from the Control Group and seven people from each community were evaluated (total of 21 individuals).

The normality analysis showed nonparametric distributions for the frequencies of MN. Despite the non-parametric distribution, we opted for the ANOVA test, assuming that the MN frequencies have a normal distribution between populations^18^. The results showed the absence of differences (p > 0.05) in the distribution of MN frequencies (Table 1), which in turn indicates the absence of mutagenic damage.

The micronucleus analysis allowed the quantification of other nuclear alterations (NA), which constitute a parameter of cytotoxic damage. The following are considered NA: “Broken Egg”, trinucleated, binucleated cells, cariorhexis, apoptosis, budding, karyolysis and nucleoplasmic bridge.

The data of NA did not show normal distribution among the groups. Thus, the Kruskal–Wallis test was used for comparing the rates of nuclear alterations among the groups. Significant differences were detected (p < 0.05) in the distribution of frequencies among groups. It was found a higher rate in the two communities studied, when compared with the control group (Table 2). However, method Dunn (Kruskal–Wallis), analysis detected similarity among the control group and the two quilombola communities groups, with no enough alterations frequency to separate it from the others.

**Table 2:** Micronucleus rates for exfoliated oral cells, collected in the control group in each studied community (7 per group, N = 21).

Micronucleus rates (n/1,000)							
Participants	Control		Urban zone		Rural zone		*p* value
	n	Rate	n	Rate	n	Rate	
1	0	0	5	0.005	15	0.015	0.057
2	3	0.003	1	0.001	11	0.011	
3	4	0.004	11	0.011	5	0.005	
4	0	0	1	0.001	2	0.002	
5	0	0	5	0.005	4	0.004	
6	2	0.002	8	0.008	3	0.003	
7	2	0.002	6	0.006	4	0.004	

*N* count number; *p* probability of differences (or effect) observed among groups being due chance; *p* value = ANOVA *p* value; Urban Zone = community quilombola of Aparecida de Goiânia city; Rural Zone = community quilombola of Silvania city. Source: Own data.

**Table 3:** Rates of other changes nuclear (including "Broken Egg”, trinucleated, binucleated cells, cariorhexis, apoptosis, budding, karyolysis and nucleoplasmic, except Micronuclei) for exfoliated buccal cells samples, collected from the control group and the two studied communities (7 per group, N = 21).

Rates of other nuclear changes (n/1,000)							
Participants	Control		Urban Zone		Rural Zone		*p* value
	n	Rate	n	Rate	n	Rate	
1	0	0	52	0.052	17	0.017	0.030
2	8	0.008	6	0.006	10	0.010	
3	6	0.006	23	0.023	17	0.017	
4	2	0.002	5	0.005	21	0.021	
5	8	0.008	27	0.027	16	0.016	
6	9	0.009	17	0.017	9	0.009	
7	12	0.012	22	0.022	11	0.011	

*N* count number, *p* probability of differences (or effect) observed between groups being due to chance; *p* value = Kruskal–Wallis *p* value; Urban Zone = community quilombola of Aparecida de Goiânia city; Rural Zone = community quilombola of Silvania city. Source: Own data.

The results found in this work suggest that the excessive cytotoxic damage caused on the study population could be inducing apoptosis^19^, so that the amount of altered cells is reduced, resulting to the lack of significance for the data of this work.

## Discussion

Genotoxicity can be caused by several factors that damage a cell and consequently can lead to mutations. To detect such genetic damage, several assays are available; including the Comet Assay and Micronucleus Test are fast, sensitive and reliable techniques that can be employed in a wide variety of cells in any organism^20^.

The studies that verify whether environmental substances affect the human population and how these changes occur are fundamental, provide information on environmental exposure and help identify potential risks to the population’s health^21^. To this end, the scientific community makes use of various cytogenetic methods, as they assess the presence and extent of damage to individuals’ genetic material^22^.

This type of study that assesses genotoxic damage is important, since according to Azqueta^23^ the human DNA is exposed to exogenous and endogenous agents that can alter its structure.

Therefore, it is relevant to study chromosomal changes, such as aneuploidy, for example, which is poorly tolerated at the cellular and organism levels. It causes proteotoxic stress and stereotyped oxidative displacement, factors that make cells sensitive to internal and environmental stress^24^.

The population is exposed to various environmental factors (chemical agents, natural sources of radiation and weather conditions) that can cause genomic instability. Additionally, lifestyle (smoking, alcoholism, drug use, diet, stress) may also be responsible for influencing genome integrity^25,26^.

The study conducted by Cuenca et al.^27^, with a Bolivian population exposed to pesticides, showed positive results for both Comet test and MN test. In addition, the results of this study demonstrate that age, alcohol consumption, and the type of water source used by participants all influence the high levels of genotoxic damage.

The data from the Comet Assay allows assessing DNA damage that can be caused by both intrinsic factors (sex, age, smoking, occupational exposure and obesity) and extrinsic factors (season, environmental exposure, diet, physical activity and alcohol consumption), a well accepted human biomonitoring test^28^. Therefore, the results of the Comet Assay in this study suggest that populations are exposed to some genotoxic factor intrinsic or extrinsic.

The data from the Comet Assay can be used to make decisions regarding public health, since it is a predictor of risk diseases, as it considers environmental, occupational or lifestyle genotoxic factors, therefore, it makes an excellent biomonitoring of the population^29^.

The analysis by Paiva et al.^30^, which used the comet assay and the chromosomal aberration test, obtained results comparatively similar to those of the present study, in a sample of rural workers from municipalities in the state of Ceará, Brazil. The study detected the association of genomic damage to the exposure of pesticide mixtures via the comet assay in peripheral blood leukocytes, however, it also reported the absence of statistical significance between the control group and the exposed groups considering the rates of chromosomal aberrations. According to Paiva et al.^30^ it is plausible that the genomic damage caused by pesticides was not sufficient to induce permanent changes or affect the formation of mitotic devices, so that the efficient performance of cell repair could explain the absence of structural and numerical chromosomal disorder in the study.

In the study conducted by Gajski et al.^31^, evaluating 200 individuals from the population with internal and external factors such as age, gender, lifestyle, seasonal variations and different meteorological parameters through the MN test, concluded that some of these factors can cause chromosomal damage in the population.

However, Gajski et al.^31^, when studying in peripheral blood lymphocytes of the general population, state that there may be interindividual variability among the subjects in the results of cytogenetic tests in relation to the aforementioned factors, fact that justifies the absence of mutagenicity found in the population researched in this study. Therefore, constant population biomonitoring studies are essential to help ensure people’s health and, consequently, their quality of life.

The genetic damage assessed by the MN test on oral cells offer a great opportunity to evaluate in a clear and precise way the appearance of genetic damage, whether it is present as a consequence of occupational or environmental risk. It can also be used to assess the genotoxic effect derived from drug use or as a result of having a chronic disease. Furthermore, the beneficial effects derived from changes in life style or taking additional supplements can also be assessed^32^. Therefore, our work supports future research aimed at evaluating the improvement in the quality of life of these populations.

## Data Availability

All data generated or analysed during this study are included in this published article.
